# Rac1 Activation Driven by 14-3-3ζ Dimerization Promotes Prostate Cancer Cell-Matrix Interactions, Motility and Transendothelial Migration

**DOI:** 10.1371/journal.pone.0040594

**Published:** 2012-07-13

**Authors:** Anna Goc, Maha Abdalla, Ahmad Al-Azayzih, Payaningal R. Somanath

**Affiliations:** 1 Clinical and Experimental Therapeutics, College of Pharmacy, University of Georgia, Augusta, Georgia, United States of America; 2 Charlie Norwood VA Medical Center, Augusta, Georgia, United States of America; 3 Department of Medicine, Georgia Health Sciences University, Augusta, Georgia, United States of America; Queensland University of Technology, Australia

## Abstract

14-3-3 proteins are ubiquitously expressed dimeric adaptor proteins that have emerged as key mediators of many cell signaling pathways in multiple cell types. Its effects are mainly mediated by binding to selective phosphoserine/threonine proteins. The importance of 14-3-3 proteins in cancer have only started to become apparent and its exact role in cancer progression as well as the mechanisms by which 14-3-3 proteins mediate cancer cell function remain unknown. While protein 14-3-3σ is widely accepted as a tumor suppressor, 14-3-3ζ, β and γ isoforms have been shown to have tumor promoting effects. Despite the importance of 14-3-3 family in mediating various cell processes, the exact role and mechanism of 14-3-3ζ remain unexplored. In the current study, we investigated the role of protein 14-3-3ζ in prostate cancer cell motility and transendothelial migration using biochemical, molecular biology and electric cell-substrate impedance sensing approaches as well as cell based functional assays. Our study indicated that expression with wild-type protein 14-3-3ζ significantly enhanced Rac activity in PC3 cells. In contrast, expression of dimer-resistant mutant of protein 14-3-3ζ (DM-14-3-3) inhibited Rac activity and associated phosphorylation of p21 activated kinase-1 and 2. Expression with wild-type 14-3-3ζ or constitutively active Rac1 enhanced extracellular matrix recognition, lamellipodia formation, cell migration and trans-endothelial migration by PC3 cells. In contrast, expression with DM 14-3-3ζ or DN-Rac1 in PC3 cells significantly inhibited these cell functions. Our results demonstrate for the first time that 14-3-3ζ enhances prostate cancer cell-matrix interactions, motility and transendothelial migration *in vitro* via activation of Rac1-GTPase and is an important target for therapeutic interventions for prostate cancer.

## Introduction

The seven members of 14-3-3 family (also known as YWHA proteins) are known to interact with more than 100 key signaling molecules in normal and cancer cells and regulate a diverse range of cellular processes [1], [2]. In general, 14-3-3 isoforms bind to two phosphorylation-dependent high affinity binding motifs such as RSXpSXP and RXXXpSXP [3], [4]. Since protein interactions with this adapter protein are dependent on the same site on protein 14-3-3 [5], why 14-3-3 is required in so many different isoforms is a long standing question. Whereas all the 14-3-3 isoforms form homodimers *in vivo*, one explanation for the requirement of different 14-3-3 proteins is to form distinct heterodimers with unique recognition motifs for specific ligands. However, while multiple 14-3-3 isoforms can form heterodimers *in vitro*, a combination of 14-3-3 ε and ζ is the only known heterodimer that is formed *in vivo* [6].

While global down-regulation of 14-3-3 expression mediates tumor suppression, expression of 14-3-3 proteins is significantly elevated in multiple cancers [7], [8], [9], [10], [11], [12]. While overexpression of 14-3-3 β and γ in NIH 3T3 cells have shown to induce oncogenic transformation [10], [13], the β, γ, ε, ζ and θ 14-3-3 gene expressions have been shown to be elevated in lung cancer alone [14]. However, of all the 14-3-3 genes, 14-3-3 σ and ζ have been most directly linked to cancer. Protein 14-3-3 σ and ζ elicit opposite effects in mammary epithelial cells [15]. Protein 14-3-3σ is thought to function as a tumor suppressor via inducing a G2-M cell cycle arrest in most cancers [16]. Its expression is down-regulated in invasive genitourinary cancers such as bladder [17], prostate [18], and the ovary [19]. In contrast, increased expression of 14-3-3ζ, a Drosophila homolog of ‘Leonardo’, has been linked to enhanced tumorigenesis and is projected as a prognostic marker and therapeutic target for cancer [20].

A correlation between increased expression of 14-3-3ζ and enhanced cell survival has been reported in prostate cancer cells [21]. Two independent studies have also indicated a relationship between elevated expression of 14-3-3ζ and incidence of prostate cancer in humans [22], [23]. However, the precise role of 14-3-3s in prostate cancer progression is largely unknown. Although the effects of 14-3-3s on proliferation, cell cycle and the survival of cancer cells are widely studied, whether or not 14-3-3s are necessary for the migration of any cancer cell types and the mechanisms leading to 14-3-3-mediated directional migration of cancer cells remains to be determined. Our previous studies in NIH 3T3 cells have shown that protein 14-3-3 over-expression results in the activation of Rac1 and p21 activated kinase (Pak) signaling pathway mediating cell migration [24]. In the current study, we investigated the specific role of 14-3-3ζ and its dimerization in the regulation of prostate cancer (PC3) cell-matrix interactions, lamellipodia formation, motility on various extracellular matrix (ECM) proteins and transendothelial migration via activation of Rac1. Our results show that inhibition of 14-3-3ζ can be a potential therapeutic strategy in prostate cancer.

## Results

### Protein 14-3-3ζ Expression Increases with Oncogenic Transformation in Prostate Cancer Cells

**Figure 1:**
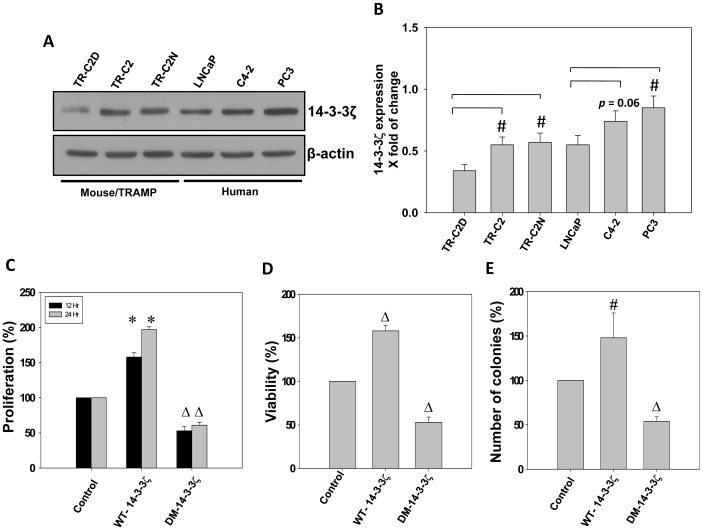
Protein 14-3-3 ζ **expression increases with oncogenic transformation in prostate cancer cells.** (A) Murine TRAMP (TR-C2D, TR-C2 and TR-C2N), human hormone responsive LNCaP as well as hormone insensitive LNCaP C4-2 and PC3 prostate cancer cell lysates were subjected for western blot comparative analysis for 14-3-3ζ expression. (B) Quantification of the above data by band densitometry analysis showing increased expression of 14-3-3ζ in prostate cancer cells compared to non-tumorigenic murine TRC2D cells. (C-E) PC3 cells were transfected with control vector, WT-14-3-3ζ and DM-14-3-3ζ and were subjected to viability (Trypan blue assay), Proliferation (MTT assay) and colony formation assay, respectively. (*p<0.001; Δ*p*<0.01; #*p*<0.05; n = 3).

Previous studies have established a relationship between elevated expression of 14-3-3ζ and increased incidence of prostate cancer in humans [22], [23]. Therefore, we sought to compare the expression levels of 14-3-3ζ among various murine (TRAMP) and prostate cancer cell lines. Our data indicated that although 14-3-3ζ is expressed in non-tumorigenic (TR-C2D) TRAMP (TRansgenic Adenocarcinoma of the Mouse Prostate) cell line, its expression is significantly higher in tumorigenic (TR-C2) and metastatic (TR-C2N) TRAMP cell lines (*p*<0.05) (Figure 1A and B). However, a significant difference in 14-3-3ζ expression between non-metastatic LNCaP and metastatic LNCaP C4-2 or PC3 cells was not observed.

It has been previously shown that 14-3-3ζ dimerization is required for its activity in cells [26]. Therefore, we next investigated whether expression and/or dimerization of protein 14-3-3ζ can mediate prostate cancer cell functions such as proliferation, viability and colony formation. Our study indicated that over-expression of wild-type protein 14-3-3ζ (WT-14-3-3ζ) in PC3 cells significantly enhanced proliferation (*p*<0.001), viability (*p*<0.01) and colony formation (*p*<0.05), compared to those expressing control vector only (Figure 1C-E). In contrast, cells expressing dimer-resistant mutant 14-3-3ζ protein (DM-14-3-3ζ), which prevents its dimerization in PC3 *cells resulted in significant reduction in proliferation (*p*<0.01), viability (*p*<0.01) and colony formation (*p*<0.01), compared to those PC3 cells expressing control vector (Figure 1C-E). Overall, these results indicate that expression and dimerization of 14-3-3ζ is necessary for prostate cancer cell function *in vitro*.

### Protein 14-3-3ζ Dimerization Drives the Rac1 GTPase Activity in PC3 Cells

**Figure 2:**
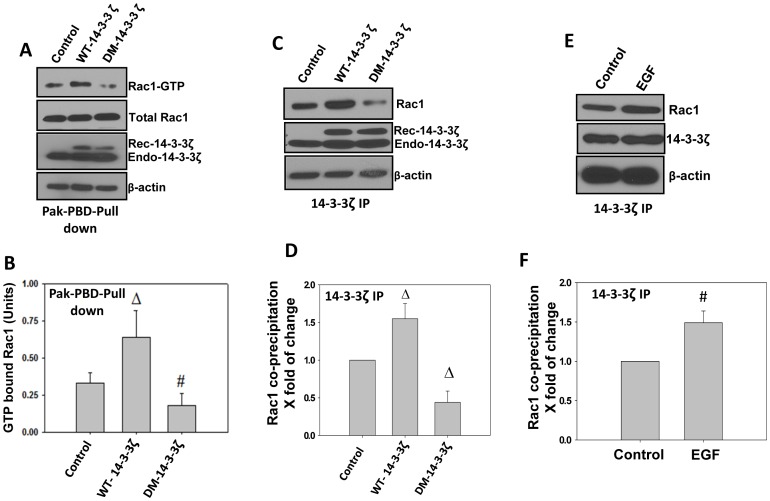
Protein 14-3-3 ζ **expression and dimerization leads to activation of Rac1 in prostate cancer cells.** (A) WT-14-3-3ζ and DM-14-3-3ζ expression plasmids along with control vector were transfected to PC3 cells and cell lysates were subjected to Rac1 activity assay using Pak-PBD-glutathione sepharose beads. (B) Quantification of the above data by band densitometry analysis showing a positive relationship between 14-3-3ζ expression, dimerization and Rac1 activity. Bars depict Rac1 activity as measured by the GTP bound Rac1 levels. (C) WT-14-3-3ζ and DM-14-3-3ζ expression plasmids along with control vector were transfected to PC3 cells and cell lysates were subjected to immunoprecipitation of 14-3-3ζ and co-precipitation of Rac1 was analyzed. (D) Quantification of the above data by band densitometry analysis. Bars depict Rac1 activity as measured by the GTP bound Rac1 levels. (E) Serum starved PC3 cells were treated with 50 µM of EGF for 12 h and lysates were subjected to immunoprecipitation of 14-3-3ζ and co-precipitation of Rac1 was analyzed. (F) Quantification of the above data by band densitometry analysis. Bars depict Rac1 activity as measured by the GTP bound Rac1 levels. (*p<0.001; Δ*p*<0.01; #*p*<0.05; n = 3).

Our previous study in NIH3T3 fibroblasts showed that protein 14-3-3 is necessary for the activation of Rac1 and p21 activated kinases 1 and 2 (Pak 1/2) [24]. In the current study, we determined whether over-expression and dimerization of protein 14-3-3ζ leads to Rac1-mediated activation of Pak1/2 in prostate cancer cells. Our study indicated that over-expression of PC3 cells with WT-14-3-3ζ significantly enhanced Rac1 activation as evidenced by the increased levels of GTP-bound Rac1, compared to control vector expressing cells (*p*<0.01) (Figure 2A and B). However, expression with DM-14-3-3ζ in PC3 cells significantly inhibited GTP-bound Rac1 levels compared to control (*p*<0.05) (Figure 2A and B). Then, we determined whether 14-3-3ζ expression and dimerization is necessary for its interaction with Rac1. Our data indicated that while immunoprecipitation of 14-3-3ζ from PC3 cells expressing WT-14-3-3ζ co-precipitated Rac1 (*p*<0.01), this interaction was abolished upon expression of PC3 cells with DM-14-3-3ζ (Figure 2C and D). Interaction between 14-3-3ζ and Rac1 in PC3 cells was also achieved with EGF (*p*<0.05) (Figure 2E and F).

**Figure 3:**
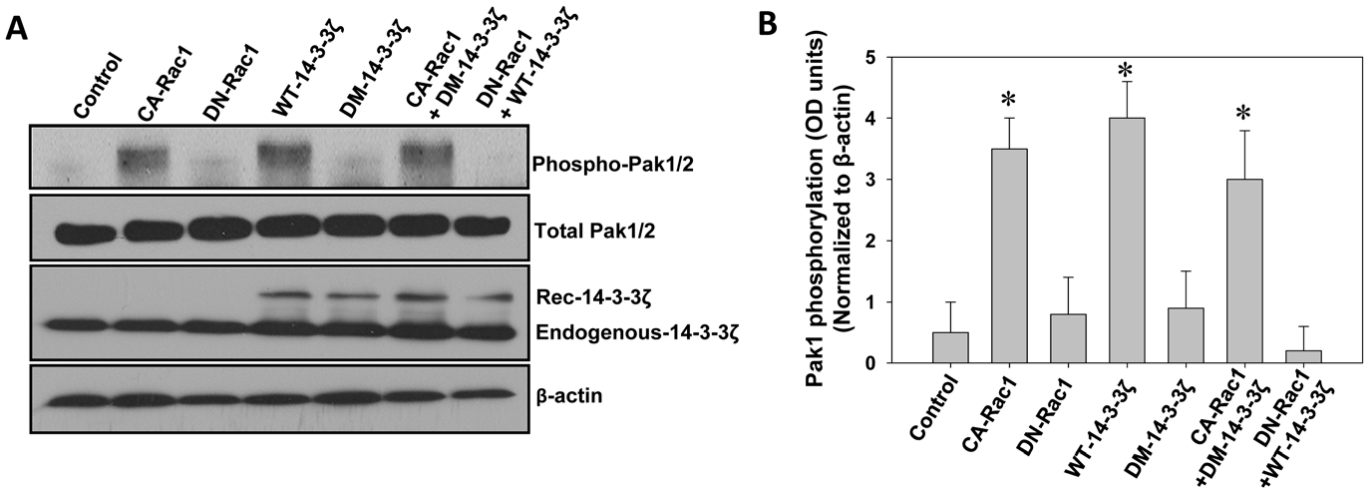
Protein 14-3-3ζ expression and dimerization leads to activation of Rac1-p21 activated kinase (Pak1) pathway in prostate cancer cells. (A) WT-14-3-3ζ, DM-14-3-3ζ, CA-Rac1 (L^61^) and DN-Rac1 (N^17^) expression plasmids and vector, alone or in combinations, were transfected to PC3 cells and cell lysates were subjected to Western analysis of phosphorylated Pak1. (B) Quantification of the above data by band densitometry analysis. Bars depict Pak1 activity as measured by the levels of phosphorylation. (*p<0.001; Δ*p*<0.01; #*p*<0.05; n = 3).

Thereafter, we analyzed whether 14-3-3ζ-Rac1 interaction can result in the modulation of Rac1-GTPase activity. In our study, over-expression of constitutively active Rac1 resulted in enhanced migration of PC3 cells on various matrix proteins. Similarly, expression with WT-14-3-3ζ also resulted in increased phosphorylation of Pak1/2 (*p*<0.001) (Figure 3A and B). Since basal levels of phosphorylated Pak1/2 in control vector transfected PC3 cells were too low, over-expression with a DN-Rac1 or DM-14-3-3ζ did not show any significant changes in Pak1/2 phosphorylation (Figure 3A and B). While co-expression of DM-14-3-3ζ with CA-Rac1 in PC3 cells showed significant increase in Pak1/2 phosphorylation compared to control vector-expressing cells (*p*<0.001), co-expression of DN-Rac1 with WT-14-3-3ζ significantly impaired Pak1/2 phosphorylation (*p*<0.001). Taken together, these results indicate that activation of Rac1 is downstream of 14-3-3ζ dimerization.

### Interaction between Protein 14-3-3ζ and Rac1 Enhances Matrix Recognition by the PC3 Cells

**Figure 4:**
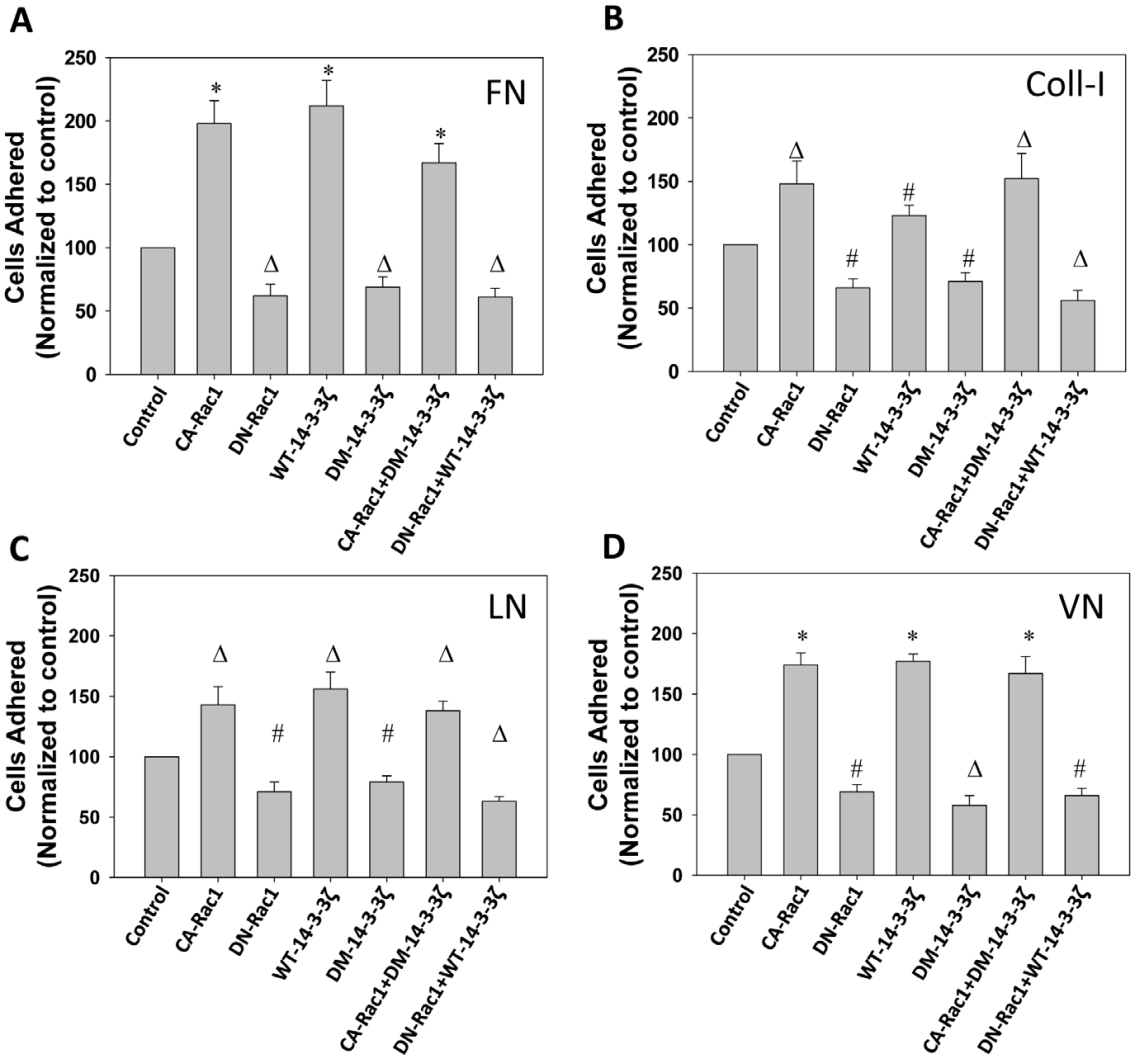
Rac1 activation is necessary for the Protein 14-3-3ζ-mediated PC3 cell-matrix interactions. (A-D) WT-14-3-3ζ, DM-14-3-3ζ, CA-Rac1 (L^61^) and DN-Rac1 (N^17^) expression plasmids and vector, alone or in combinations, were transfected to PC3 cells and serum starved cells were subjected to cell adhesion assay on extracellular matrix proteins such as Fibronectin (FN), Collagen type I (COLL-1), Laminin (LN) and Vitronectin (VN), respectively, with a cell density of 1×10^4^ cells/well in the presence of 10% FBS. Bars depict number of PC3 cells adhered to specific ECM proteins. (*p<0.001; Δ*p*<0.01; #*p*<0.05; n = 3 of quadruplicates).

Since Rac-GTPases are master regulators of cytoskeletal remodeling in response to cues from the extra-cellular microenvironment, we studied whether modulation of protein 14-3-3ζ-Rac1 signaling in prostate cancer cells will have any effect on their ability to recognize and bind to ECM proteins such as fibronectin (FN), vitronectin (VN), collagen I (COLL-I) and laminin (LN), which are abundantly expressed in various tissues harboring prostate cancer cells. Our study indicated that while expression with WT-14-3-3ζ and CA-Rac1 enhanced PC3 cells adhesion to these ECM proteins, expression with DM-14-3-3ζ or DN-Rac1 significantly impaired this process (Figure 4A-D). While impaired adhesion by DM-14-3-3ζ expression was rescued by co-expression with CA-Rac1, co-expression of WT-14-3-3ζ in DN-Rac1 expressing PC3 cells did not rescue the impaired cell adhesion (Figure 4A-D), indicating that 14-3-3ζ acts upstream of rac1 activation in the regulation of PC3 cell-matrix interactions.

### Protein 14-3-3ζ-Rac1 Signaling Regulates Lamellipodia Formation in PC3 Cells

**Figure 5:**
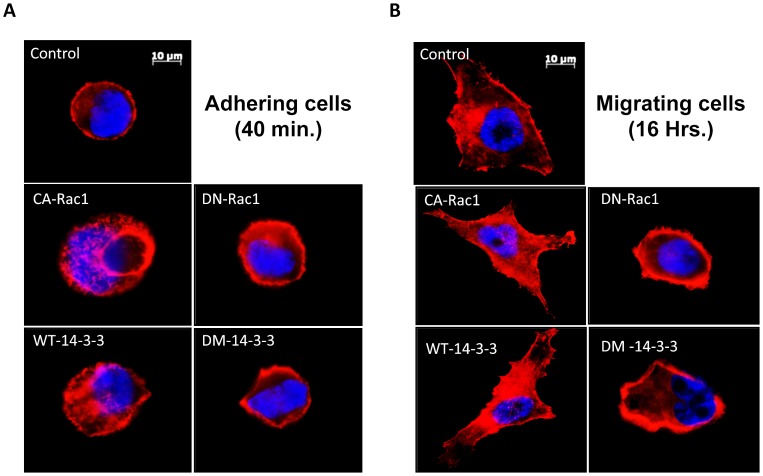
Protein 14-3-3 ζ**-Rac1 cooperation regulates lamellipodia formation in PC3 cells.** (A-B) WT-14-3-3ζ, DM-14-3-3ζ, CA-Rac1 (L^61^) and DN-Rac1 (N^17^) expression plasmids and vector, alone or in combinations, were transfected to PC3 cells, plated on cell culture chambers in the presence of 10% FBS and fixed at 40 minutes (A) or 16 hours (B) after plating. Paraformaldehyde fixed cells were stained with Alexa Fluor (Red) labeled phalloidin to detect newly polymerized actin cytoskeleton and lamellipodia.

Our further analysis using phalloidin staining of PC3 cells that specifically stains newly polymerized actin cytoskeleton indicated that over-expression with WT-14-3-3ζ or CA-Rac1 resulted in enhanced lamellipodia formation in newly spreading cells compared to control vector expressing untreated PC3 cells in the presence of 10% FBS (Figure 5A). In contrast, expression with DM-14-3-3ζ or DN-Rac1 resulted in reduced lamellipodia formation compared to control cells (Figure 5A). Similar effects were also observed in migrating PC3 cells analyzed for phalloidin staining. PC3 cells expressing WT-14-3-3 or CA-Rac1exhibited enhanced lamellipodia formation compared to vector-expressing cells (Figure 5B). Together, these results indicate that 14-3-3ζ-Rac1 association is involved in lamellipodia formation in prostate cancer cells.

### Cooperation between Protein 14-3-3ζ and Rac1 Enhances EGF-stimulated PC3 Cell Directional Migration and Transendothelial Migration

**Figure 6:**
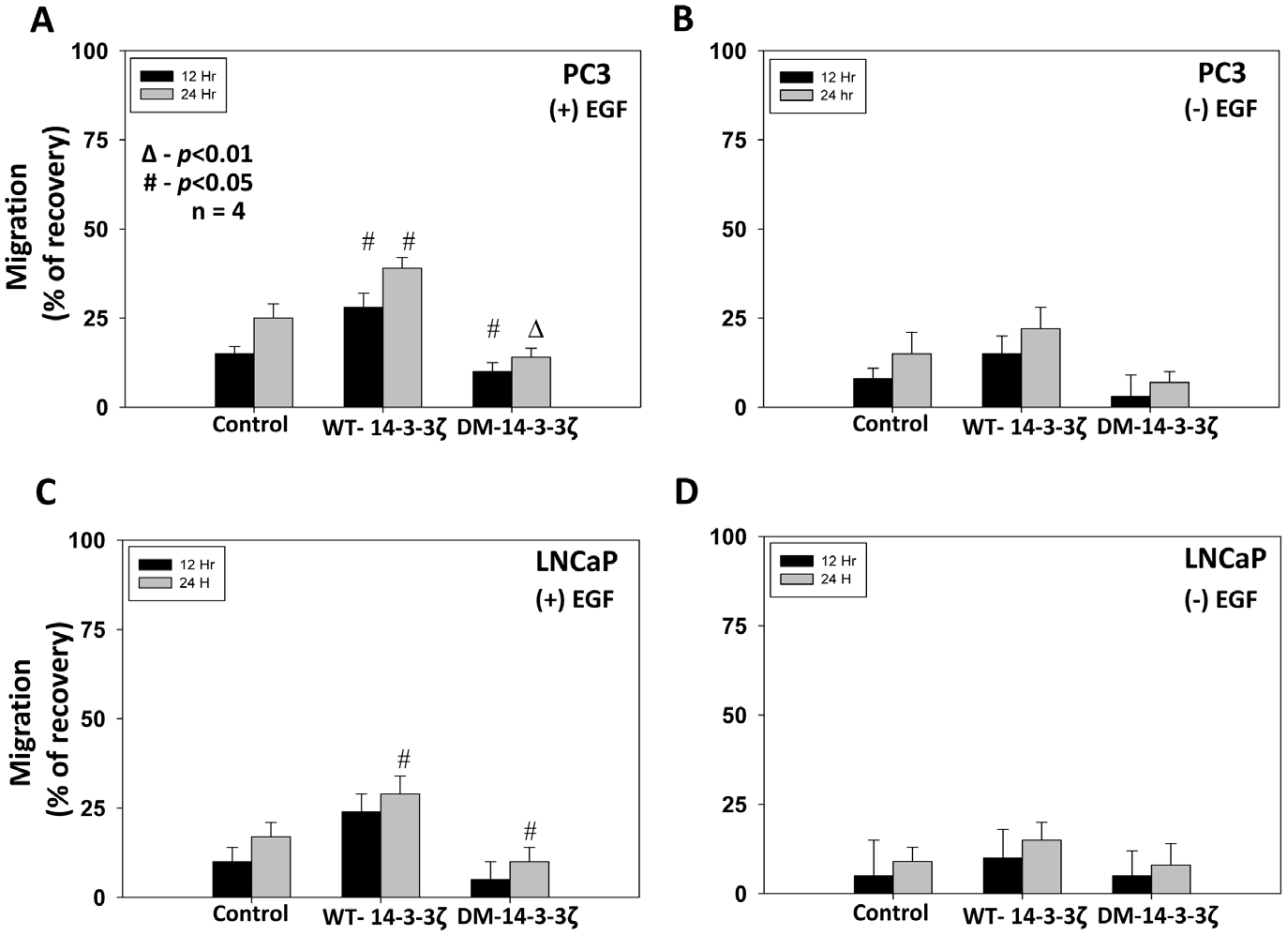
Protein 14-3-3ζ dimerization is necessary for **LNCaP and PC3 cell migration.** (A-D) WT-14-3-3ζ and DM-14-3-3ζ expression plasmids or control vector were transfected to LNCaP and PC3 cells. Cells were then subjected to cell migration assay through a scratch made in the monolayer of cells. Bars depict migration of PC3 cells as measured by its scratch recovery. (*p<0.001; Δ*p*<0.01; #*p*<0.05; n = 4 of quadruplicates).

Next, we examined whether 14-3-3ζ plays a role in prostate cancer cell migration. To do this we transfected androgen-responsive LNCaP cells and androgen-insensitive PC3 cells with WT-14-3-3ζ and DM-14-3-3ζ and subjected them for migration assay in 24 well plates in the presence and absence of EGF. Our results showed that while expression of both LNCaP and PC3 cells with WT-14-3-3ζ significantly enhanced cell migration compared to vector control expressing cells (*p*<0.05) in the presence of EGF, expression with DM-14-3-3ζ resulted in impaired cell migration after 24 h (*p*<0.01 for PC3 cells and *p*<0.05 for LNCaP cells) (Figure 6A and C). Although a similar trend was observed in the absence of EGF, the data was not statistically significant (Figure 6B and D).

**Figure 7:**
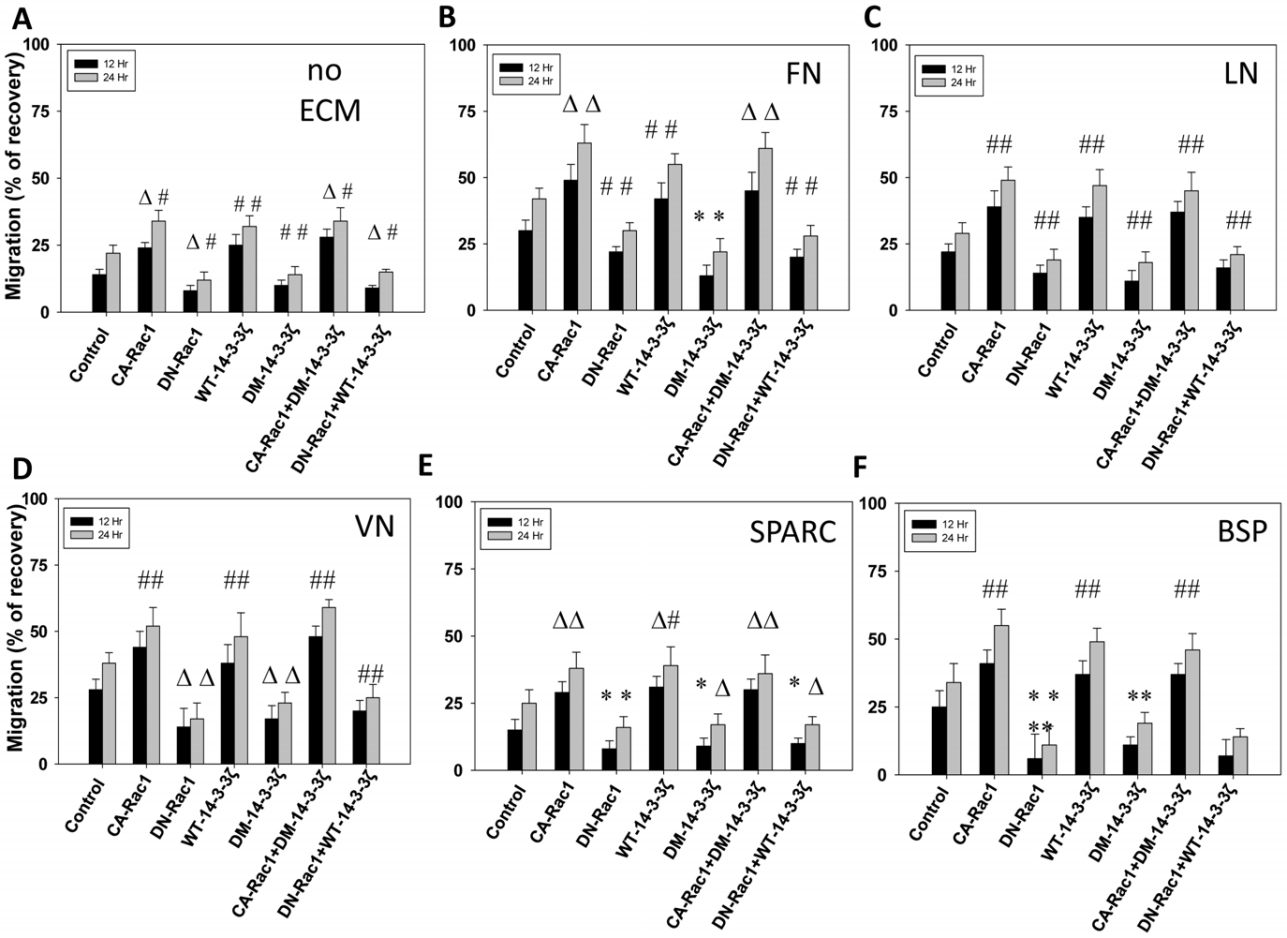
Rac1 activation is necessary for the Protein 14-3-3 ζ**-mediated PC3 cell migration.** (A-F) WT-14-3-3ζ, DM-14-3-3ζ, CA-Rac1 (L^61^) and DN-Rac1 (N^17^) expression plasmids and vector, alone or in combinations, were transfected to PC3 cells and cells were subjected to cell migration assay on plastic or extracellular matrix proteins such as Fibronectin (FN), Laminin (LN) and Vitronectin (VN), Osteonectin (SPARC) and Bone sialoprotein (BSP; Osteopontin), respectively. Bars depict migration of PC3 cells on various ECM proteins as measured by its scratch recovery. (*p<0.001; Δ*p*<0.01; #*p*<0.05; n = 4 of quadruplicates).

**Figure 8:**
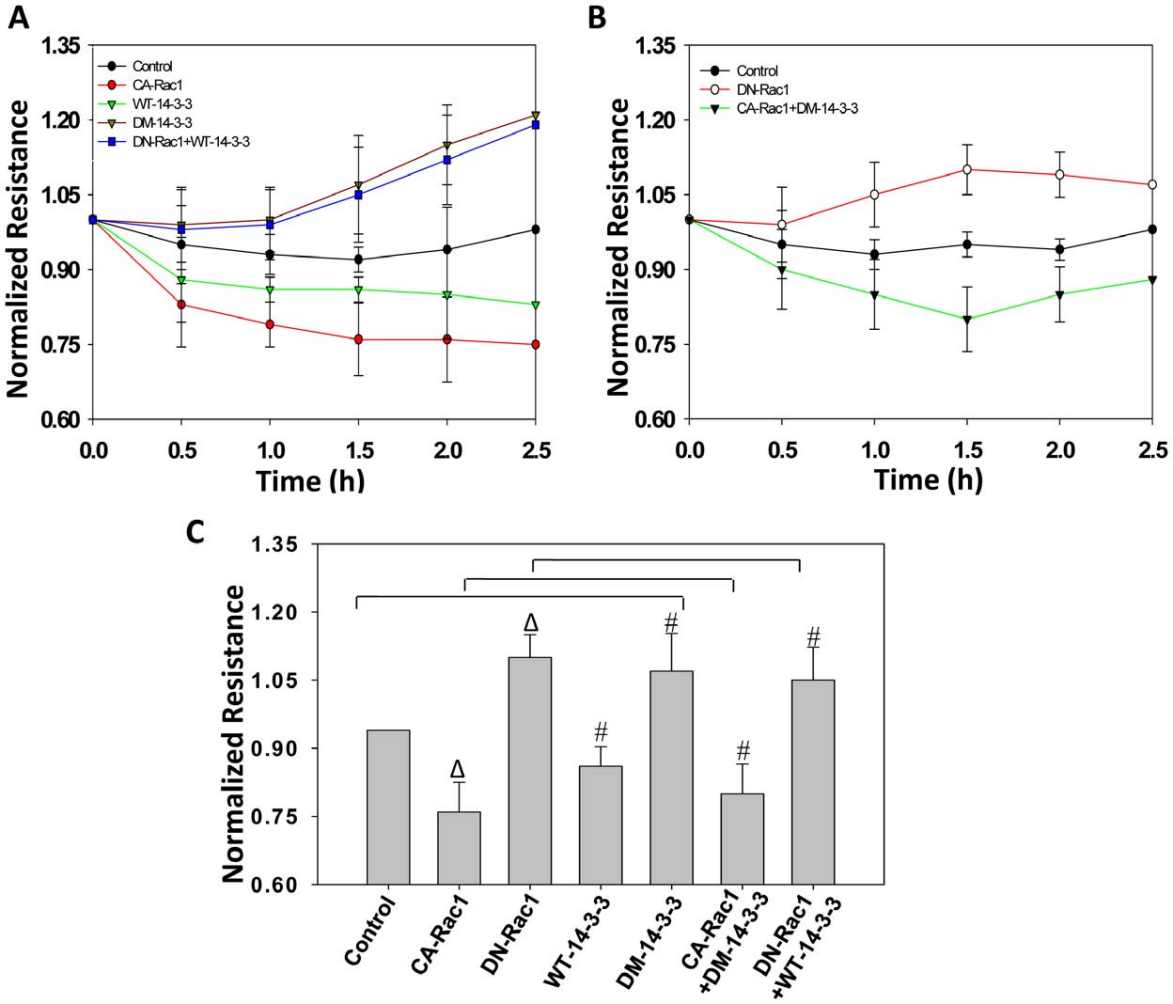
Protein 14-3-3 ζ**-mediated Rac1 activation regulates PC3 cell transendothelial migration.** (A-B) WT-14-3-3ζ, DM-14-3-3ζ, CA-Rac1 (L^61^) and DN-Rac1 (N^17^) expression plasmids, vector control as well as a combination of DN-Rac1 and WT-14-3-3ζ, were transfected to PC3 cells and cells were subjected to transendothelial migration assay *in vitro* using ECIS. (C) Analysis of transendothelial migration of PC3 cells at 1.5 h after introduction of cells to endothelial monolayer in ECIS array chips demonstrating the role of 14-3-3ζ dimerization on activation of Rac1 in mediating transendothelial migration of PC3 cells (Δ*p*<0.01; #*p*<0.05; n = 5).

Next we determined if 14-3-3ζ-Rac1 cooperation is necessary for the transendothelial migration as well as directional migration of PC3 cells in response to EGF on various ECM proteins that are abundant in tissues harboring prostate cancer cells during the different stages of tumor growth, invasion, transendothelial migration and metastasis to tissues such as bone. Our analysis indicated that while expression with WT-14-3-3ζ and CA-Rac1 significantly enhanced the PC3 cell migration on ECM proteins such as FN, VN, LN, Bone sialoprotein (BSP; Osteopontin) and SPARC (Osteonectin), expression with DM-14-3-3ζ or DN-Rac1 significantly impaired directional migration of PC3 cells (Figure 7A-F). Similarly, while expression with WT-14-3-3ζ or CA-Rac1 significantly enhanced transendothelial migration of PC3 cells, expression with DM-14-3-3ζ or DN-Rac1 significantly impaired transendothelial migration by PC3 cells (Figure 8A-C). Whereas impaired PC3 cell migration by DM-14-3-3ζ expression was rescued by co-expression with CA-Rac1, impaired motility and transendothelial migration by DN-Rac1 expressing PC3 cells was not rescued by co-expression with WT-14-3-3ζ (Figure 8A-C). Collectively, our results demonstrate the role of 14-3-3ζ in mediating Rac1 activation necessary for PC3 cell migration and transendothelial migration.

## Discussion

The primary observation from our current study is that expression with protein 14-3-3ζ in PC3 cells results in enhanced cell-ECM interactions, lamellipodia formation, cell migration and transendothelial migration via activation of Rac1-GTPase. We previously reported that over-expression of protein 14-3-3β results in enhanced Rac1 and Pak activity in NIH 3T3 cells, resulting in enhanced integrin activation, lamellipodia formation, cell migration and extracellular matrix assembly [24]. We have also shown that activation of Rac1-Pak1/2 pathway is involved in oncogenic transformation of Rat-1a cells [25], implying that similar mechanism by 14-3-3ζ in cancer cells may enhance tumor growth, transendothelial migration and metastasis. In the current study, we observed that expression with WT-14-3-3ζ in PC3 cells resulted in enhanced adhesion and migration on various ECM proteins, which are abundant in tissues that harbor prostate cancer cells during the several stages of tumor cell invasion, trans-vascular migration and bone metastasis. However over-expression of a mutant of 14-3-3ζ (DM-14-3-3ζ), which has previously been shown to prevent its dimerization [26], in PC3 cells did not exhibit these effects, instead resulted in impaired cell adhesion and migration compared to control PC3 cells. While WT-14-3-3ζ expression resulted in enhanced lamellipodia formation and increased activity of Rac1-GTPase as evidenced by the enhanced phosphorylation of Pak1/2, expression with DM-14-3-3ζ opposed these effects. Further, our data indicated that dimerization of protein 14-3-3ζ is necessary for the activation of Rac1, which in turn, regulates motility and transendothelial migration of prostate cancer cells. Altogether, our study shows a direct association between protein 14-3-3ζ and Rac1 in the regulation of ECM interaction with PC3 cells, their motility and transendothelial migration, and that preventing dimerization of 14-3-3ζ can inhibit these effects.

Although the role of 14-3-3 proteins in cell survival and proliferation has been extensively studied, the specific role of 14-3-3s in the regulation of cell-matrix interactions, motility and transendothelial invasion and the molecular mechanisms regulating the process is not clearly understood. Cell-matrix interactions and migration are the key properties of the metastatic cancer cells, and the first step in this process involves cytoskeletal remodeling, mainly controlled by small Rho GTPases [27].Two independent studies identified protein kinase D (PKD) as a candidate protein associated with F-actin formation and interacts with 14-3-3 in the regulation of cytoskeletal assembly [28], [29]. However, PKD was involved in a negative regulation of active cytoskeletal remodeling in the leading edge with over-expression of constitutively active PKD resulting in impaired cell migration via activation of RhoA. Other mechanisms that have been implicated in the 14-3-3-mediated cell migration include its involvement in inactivation of cortactin, an actin binding protein, via PKD-mediated phosphorylation at Ser298, leading to inhibition of cell migration [30].

Studies during the last decade have revealed the importance of 14-3-3 proteins in the positive regulation of cell migration involving Rac1-GTPases. Initial evidence of 14-3-3 and Rac1 association was first reported in a CHO cell model [31]. Our lab has previously shown that 14-3-3β activates Rac1 and Pak1 signaling in the regulation of NIH 3T3 cell-matrix interaction, migration and extracellular matrix assembly via affinity modulating of integrin α_5_β_1_ [24]. We also showed that expression with 14-3-3β results in the translocation of Rac1 to the membrane ruffles enhancing Pak1 activity and lamellipodia formation in NIH3T3. Following this, a more recent study indicate that 14-3-3 controls cell mechanics and cytokinesis in *Dictyostelium* via coordination of Rac1 activity, myosin II and microtubules [32]. However, there are no other reports on the association of 14-3-3 and Rac1-GTPases in the regulation of cell migration of any cancer cells. In the current study, our results indicate that over-expression of WT-14-3-3ζ results in lamellipodia formation in spreading and migrating PC3 cells similar to constitutively active Rac1 expressing cells. In contrast, DM-14-3-3ζ inhibited lamelipodia formation. Our results from Rac1 activity assay and phosphorylation of Pak1/2, a downstream substrate of Rac-GTPases, both indicated that 14-3-3ζ activates Rac1. Furthermore, the fact that co-expression with DM-14-3-3ζ did not inhibit the phosphorylation of Pak1/2 mediated by expression with CA-Rac1 indicate that 14-3-3ζ dimerization is upstream of Rac1 activation. Although the exact mechanism by which 14-3-3ζ regulates Rac1 activity in prostate cancer cells is not evident from our results, a possible mechanism would be the interaction of 14-3-3ζ with one of its guanine nucleotide exchange factors (GEF) and its delivery to Rac1 resulting in its activation.

Among the 14-3-3 isoforms, enhanced expression of 14-3-3ζ has been implicated to develop tumor resistance to chemotherapy drugs, including prostate cancer [21], [33]. In contrast, down-regulation of 14-3-3 expression sensitizes cancer cells to chemotherapeutic drugs [2], [20], including prostate cancer [21], implicating the therapeutic potential of 14-3-3ζ for cancer therapy. Recent advances in SiRNA based knockdown of 14-3-3 proteins as well as peptide inhibitors preventing dimerization of 14-3-3 such as R18 (difopein, a dimer of R18) [34] or small molecule compounds such as FOBISIN 101 [35] shows the promise of developing anti-14-3-3 therapy for cancer.

In conclusion, we identify 14-3-3ζ and Rac1 as novel partners in the pathway regulating prostate cancer cell-matrix interactions, motility in response to invasive and metastatic stimuli as well as for intravasation of prostate cancer cells. Our results indicate that 14-3-3 is a potential target for therapeutic interventions in prostate cancer.

## Methods

### Reagents, Cell Lines and Antibodies

Human PC3, LNCaP and LNCaP C4-2 cells (ATCC, Manassas, VA) were used and maintained in DMEM-High glucose (HyClone, Thermo Scientific, Logan, UT) with 10% fetal bovine serum, 100 units/ml penicillin, and 100µg/ml streptomycin in a 5% CO_2_ atmosphere at 37°C. Murine TRAMP (TR-C2D, TR-C2 and TR-C2N) cells were gifted by Dr. Barabara Foster, Baylor College of Medicine, TX. Primary antibodies against phospho-Pak1/2 Ser144/142, 14-3-3ζ and pan-protein-14-3-3 were purchased from Cell Signaling (Boston, MA). Anti-Rac1 antibodies, fibronectin and laminin were obtained from BD Biosciences (Franklin Lakes, NJ). Primary antibodies against β-actin were purchased from Sigma, St. Louis, MO. All secondary antibodies were obtained from BioRad (Hercules, CA). Alexa Fluor555-labeled phalloidin was purchased from Invitrogen (Carlsbad, CA). Osteopontin (Bone sialoprotein), osteonectin (SPARC) and vitronectin were purchased from R&D Systems (Minneapolis, MN).

### Transient Transfections

Human PC3 cells were transiently transfected with pEBG plasmids WT-14-3-3ζ (GST tagged), DM-14-3-3ζ (dimerization-resistant 14-3-3ζ-GST tagged; 14-3-3 E5K, L12AE to Q12QR, Y82Q, K85N, and E87Q), CA-Rac1 (Rac1-L^61^), and DN-Rac1 (Rac1-N^17^) constructs, respectively, or in combinations of WT-14-3-3ζ with DN-Rac1 (Rac1-N^17^) constructs and DM-14-3-3ζ with CA-Rac1 (Rac1-L^61^), using lipofectamine 2000 Invitrogen (Carlsbad, CA) transfection reagent according to the manufacturer's protocol. Protein 14-3-3 constructs, originally generated by Joseph Avruch lab, Massachusetts General Hospital, Boston were obtained from Addgene, Cambridge, MA. Control cells were transiently transfected with empty vector pBabe-Puromycin. Approximately 70–80% transfection efficiency was obtained.

### Rac1 Activation Assay

Rac1 activity was measured using a Rac activity assay kit from Cytoskeleton (Denver, CO) according to manufacturer's protocol. Briefly, the PBD domain of PAK fused to glutathione *S*-transferase (GST) and conjugated with glutathione-Sepharose 4B beads were mixed with 1 mg of total protein in lysate from PC3 cells transiently transfected with WT-14-3-3ζ, DM-14-3-3ζ, CA-Rac1, or DN-Rac1 plasmids or in combinations of CA-14-3-3ζ with DN-Rac1 and DM-14-3-3ζ with CA-Rac1, followed by incubation at 4°C for 1 h. Beads were collected by centrifugation and were washed three times with washing buffer with 1X Complete protease inhibitors (Roche Applied Science, Indianapolis, IN) and twice with 1X PBS. Proteins were eluted by boiling beads in 2X Laemmli sample buffer (BioRad, Hercules, CA) for 5 min, separated on a 12% SDS–polyacrylamide gel and blotted with Rac1 antibodies.

### Trypan Blue Viability Assessment

Cells were grown to confluence in DMEM with 10% FBS. The cells were plated in 96-well plates at a density of 5×10^3^ cells/100 µl in DMEM and allowed to adhere overnight. Following this, cells were transfected with respective plasmids and cultured for 48 h. Cells were then cultured for an additional 24 h in serum free conditions. Cells were then harvested by trypsinization, stained with a trypan blue solution (0.04% w/v, Invitrogen, Carlsbad, CA), and counted using the hemocytometer. Total cells and trypan blue-stained (i.e., nonviable) cells were counted, and the percentage of nonviable cells was calculated.

### Cell Proliferation Assay

Proliferation of PC3 cell lines was determined using the nonradioactive BrdU-based cell proliferation assay (Roche Applied Science) according to the manufacturer's protocol. Briefly, transfected PC3 cells were plated in 96-well flat bottom plates at a density of 5 × 10^3^ cells per 100 µl, and allowed to grow for 48 h. Cells were then cultured for an additional 24 h in serum free conditions. PC3 Cells were then subjected to a 5-bromo-2-deoxyuridine assay using the BrdU Labeling and Detection Kit III (Roche Applied Science) according to the manufacturer's protocol. BrdU incorporation into the DNA was determined by measuring the absorbance at both 450 and 690 nm on an ELISA plate reader. The data are presented as mean ± S.D.

### Colony Formation Assay

Colony formation assay was performed using the standard protocol [36]. In this approach, PC3 cells after transfections were cultured on six-well plates until the monolayer was reached. After seven days of culture in serum containing medium, each of the wells was counted for the number of colonies, and WT-14-3-3ζ and DM-14-3-3ζ expressing cells were compared with the vector-expressing control. Plates were fixed using 2% paraformaldehyde, briefly stained with crystal violet, and counted visually or by using ImageJ software. The data are presented as mean ± S.D.

### Cell Adhesion Assay

Cell adhesion assays were performed as previously described [37]. Briefly, PC3 cells were suspended in DMEM High glucose medium. The cells in suspension were immediately transferred to ECM protein-coated 12-well plates at a density of 1×10^5^ cells/well. After incubation at 37°C for 45 min, the wells were washed three times with 1X PBS. Cells were then fixed in 1% paraformaldehyde, stained with 0.05% crystal violet, examined under the microscope and adherent cells were quantified using NIH Image J software.

### Migration Assay

Migration assays were performed as previously described [38]. Briefly, LNCaP and PC3 cells, after transient transfections were grown on 12-well plates on specific ECM proteins (1 µg/ml) to reach confluence (approximately 8 h) and then serum starved for 3 h. A scratch was made in the monolayer and pictures were taken at 0, 12 and 24 h. The rate of migration (as measured by scratch recovery) in the presence of 10% FBS was calculated using the following equation (1-T_t_/T_0_) X 100, where T_t_ is the area at the endpoints and T_0_ is the area at time zero.

### Immunocytochemistry

The immunofluorescence staining was performed as described previously [24]. Briefly, PC3 cells, after transient transfections, were plated on cell culture chamber slides with a cell density of 1×10^4^ cells/well in the presence of 10% FBS (Fisher scientific, Pittsburgh, PA) followed by fixation at different time points with 1% paraformaldehyde in PBS. Cells were permeabilized with 0.1% triton X-100 in PBS. The non-specific staining was blocked with 2% BSA for 1 h at room temperature. The fixed and permeabilized cells were incubated with standardized dilution of Alexa Fluor555-labeled phalloidin for 40 min and washed. The slides were mounted with Vectashield (Vector Laboratories). The images were taken by Zeiss fluorescent microscope. Random fields were examined for cell morphology. Characteristic features applicable to more than 70% of the cell population were considered as the morphology of the cell with respect to its transfection status.

### Transendothelial Migration Assay

Transendothelial migration of prostate cancer (PC3) cell lines was measured using Electric Cell-substrate Impedance Sensing (ECIS) equipment with human dermal microvascular endothelial cells (ATCC, Manassas, VA) plated on 8W10E+ array chips (Applied Biophysics, Troy, NY). Following this, control PC3 as well as transfected cells were directly added onto endothelial cell monolayer at a density of 5×10^4^ cells per well in 100 µl medium. To detach the cells from plate, cell dissociation buffer (20 mM EDTA in PBS, pH 7.4) was used to avoid integrin/receptor loss due to trypsin digestion. Real-time measurements of the transendothelial migration of PC3 cells were recorded by the ECIS up to 12 h.

### Western Analysis

Whole cell lysates were prepared using lysis buffer [50 mM Tris-HCl (pH = 7.4), 1% TritonX-100, 150 mM NaCl, 1 mM EDTA, 2 mM Na_3_VO_4_, and 1X Complete protease inhibitors (Roche Applied Science, Indianapolis, IN)]. The protein concentration was measured by the D_c_ protein assay (Bio-Rad, Hercules, CA). Western analyses were performed using standard Laemmle's method as done previously [37]. Densitometry was done using NIH Image J software.

### Statistical Analysis

All the data are presented as means ± SD. To determine significant differences between treatment and control values, we used the Student’s two-tailed *t* test. The significance was set at 0.05 levels (marked with symbols wherever data are statistically significant).
